# Americans Use Medicines

**Published:** 1898-09

**Authors:** 


### Americans Use Medicines.

Prof. Ulrich says the American people are a great medicine-taking nation. If the quantity of drugs prescribed by physicians, the masses of patent medicines, the barrels of so-called home remedies, such as teas, decoctions, infusions and other monstrosities, swallowed by the American people were ascertained, collected, arranged, and published in a book, it would strike the reader dumb with astonishment.—*Practical Druggist.*

